# Comparison of the effects of intramyocardial and intravenous injections of human mesenchymal stem cells on cardiac regeneration after heart failure

**DOI:** 10.22038/ijbms.2020.40886.9660

**Published:** 2020-07

**Authors:** Behnaz Mokhtari, Nahid Aboutaleb, Donya Nazarinia, Mahin Nikougoftar, Seyed Mohammad Taghi Razavi Tousi, Mohammad Molazem, Mohammad-Reza Azadi

**Affiliations:** 1 Physiology Research Center, Iran University of Medical Sciences, Tehran, Iran; 2 Department of Physiology, Faculty of Medicine, Iran University of Medical Sciences, Tehran, Iran; 3 Blood Transfusion Research Center, High Institute for Research and Education in Transfusion Medicine, Tehran, Iran; 4 Medical Biotechnology Research Center, Guilan University of Medical Sciences, Rasht, Iran; 5 Department of Veterinary Diagnostic Imaging, Faculty of Veterinary Medicine, University of Tehran, Tehran, Iran

**Keywords:** Heart function, Intramyocardial injection, Intravenous injection, Isoproterenol-induced heart, failure, Mesenchymal stem cells

## Abstract

**Objective(s):**

Existing studies have demonstrated that intravenous and intramyocardial-administrated mesenchymal stem cells (MSCs) lead to tissue repair after cardiac disorders. We compared the efficiency of both administration methods.

**Materials and Methods:**

A rat model of isoproterenol-induced heart failure (ISO-HF) was established to compare the effects of intravenous and intramyocardial-administrated MSCs on cardiac fibrosis and function. The animals were randomly assigned into six groups: i) control or normal, ii) ISO-HF (HF) iii) ISO-HF rats treated with intramyocardial administration of culture medium (HF+IM/CM), iv) ISO-HF rats treated with intravenous administration of culture medium ( HF+IV/CM), v) ISO-HF rats treated with intravenous administration of MSCs (HF+IV/MSCs), vi) ISO-HF rats treated with intramyocardial administration of MSCs ( HF+IM/MSCs). Cultured MSCs and culture medium were administrated at 4 weeks after final injection of ISO. Heart function, identification of MSCs, osteogenic differentiation, adipogenic differentiation, cardiac fibrosis and tissue damage were evaluated by echocardiography, flow-cytometery, von Kossa, oil red O, Masson’s trichrome and H & E staining, respectively.

**Results:**

Both intravenous and intramyocardial MSCs therapy significantly improved heart function and reduced cardiac fibrosis and tissue damage (*P<*0.05), whereas the cultured medium had no beneficial effects.

**Conclusion:**

In sum, our results confirm the validity of both administration methods in recovery of HF, but more future research is required.

## Introduction

Heart failure (HF) is a developed end-stage cardiac disorder and a common terminal outcome of heart overload and myocardial injury (1). HF can result in reduced and insufficient blood supply of body metabolic requirements (2, 3). HF is associated with increasing urbanization, changes in life style and environmental conditions and has posed an ever-increasing challenge because of its high rate of morbidity and mortality around the world (4). HF is characterized by reduced ejection fraction (EF), ventricular hypertrophy, contraction of the left ventricle (LV), abnormalities in active relaxation, cardiac fibrosis and ventricular remodeling (5). Although many treatment options such as beta blocker, diuretics, digoxin, and angiotensin converting enzyme inhibitors have been shown to cause symptom relief, rate of morbidity and mortality remains high (6). It is well-documented that cell transplantation could ameliorate cardiac fibrosis in the site of injury in HF (7, 8). Subcutaneous injection of isoproterenol (ISO) is able to enhance myocardial destruction, irreversible loss of a large number of cardiomyocytes that result in the extent of myocardial fibrosis and chronic HF (9). The presence of an intact vascular system in ISO-induced heart failure (ISO-HF) makes it a good experimental model to investigate ventricular fibrosis and HF. During pathological condition, oxidative stress can give rise to extension of cell death (10-12). Human mesenchymal stem cells (hMSCs) are multipotent and undifferentiated cells, capable to differentiate into various cell types such as chondrocytes, osteoblasts, cardiac myocytes, and adipocytes (13, 14). The pro-angiogenic properties of MSCs make them promising candidates for restoration of endothelial function in HF (15, 16). MSCs have been found to secrete a wide range of factors that can affect numerous physiological and pathophysiological processes such as tissue healing, collagen deposition, angiogenesis, apoptosis, adverse LV remodeling and mitochondrial function (17-20). There are several methods for administration of MSCs in the site of injury in various cardiac disorders. These include intravenous, intramyocardial, intracoronary and transendocardial administrations. There are controversial findings for administration methods of MSCs in various cardiac disorders (21). Many studies are based on the direct injection in the site of injury because it is usually believed that intravenous-administrated MSCs are poorly engrafted in damaged myocardium (22). These studies show that intramyocardial administration of stem cells in damaged myocardium provides beneficial effects because MSCs can directly differentiate into functioning myocytes or secrete some factors to increase myocardial repair (23). On the other hand, some reports have shown that intravenous administration of MSCs exerts anti-inflammatory effects and has several advantages such as possibility of repeated injection of MSCs and subsequent sustained therapeutic effects. For example, in a recent study, Luger D. *et al.,* reported that intravenously-administered MSCs after acute myocardial infarction (MI) ameliorated post-ischemic adverse remodeling and the progressive deterioration in LV function. Moreover, they found that MSCs contributed to the suppression of acute inflammatory responses through decreasing the number of neutrophils in heart and natural killer (NK) cells in the heart and spleen (24). In the present study, we examined both intravenous and intramyocardial strategies for administration of MSCs to the site of injury after ISO-HF to compare their efficiency in reduction of fibrosis and improvement of heart function. 

## Materials and Methods

***Animals***

In this experimental study, six-week-old male Wistar rats weighing 200±20 g were purchased from animal laboratory of Iran University of Medical Sciences. Rats were maintained in a temperature- and light-controlled room, at 12 hr: 12 hr light- dark cycle, and moisture (40–60%) and allowed to obtain standard rat chow and water *ad libitum*. The animals were placed five per cage to maintain social interaction.

***Experimental design***

Animals were assigned into six groups: a normal group of healthy animals (control; n=7), ISO-HF group (HF, n=7), ISO-HF rats treated with intramyocardial administration of culture medium (CM) (HF+IM/CM; n=7), ISO-HF rats treated with intravenous administration of CM (HF+IV/CM; n=7), ISO-HF rats treated with intramyocardial administration of MSCs (HF+IM/MSCs; n=7), and ISO-HF rats treated with intravenous administration of MSCs (HF+IV/MSCs; n=7). Preparation of conditioned medium has been described in our previous studies (25-27), whereas culture medium is comprised from Dulbecco′s Modified Eagle′s Medium (DMEM) supplemented with 10% fetal bovine serum (FBS). 

***HF model***

Previous reports have shown that excessive administration of catecholamines leads to extensive destruction of myocardium and fibrosis (28). To induce infarct-like myocardial necrosis, 170 mg/kg ISO (dissolved in 0.5 ml saline) was subcutaneously administered into animals once daily for 4 days. At 4 weeks after final injection of ISO, transthoracic 2D guided M-mode echocardiography was performed to approve induction of HF. 

***Isolation and transplantation of MSCs***

The hMSCs were isolated from human amniotic membrane based on our previous study (29). In brief, amniotic membrane was dissected from decidual tissue. After several washing in phosphate-buffered saline (PBS), amniotic membrane was immediately transferred to the laboratory. After removing blood clots and vessels, amniotic membrane was divided into small pieces through a mechanical method. Then, the samples were centrifuged at 1250 rpm for 5 min. After removing supernatant, 30 ml of collagenase was added to the pellet. The pellet was maintained at 37 ^°^C in a humidified 5% CO_2_ incubator for 1 hr, then samples were centrifuged at 1250 rpm for 5 min again. After removing supernatant, trypsin (0.25% containing 1 ml EDTA) was added to the pellet. The pellet was maintained at 37 ^°^C in a humidified 5% CO_2_ incubator for 30 min again and the washing procedure was repeated twice more. To remove red blood cells (RBCs), the resulting pellet was treated with Tris-ammonium chloride for 10 min. Afterwards, the resulting pellet containing MSCs was plated in DMEM supplemented with 10% FBS. After 24 hr of plating, medium culture was removed to omit non adherent cells. The adherent cells were propagated for three to four passages prior to transplantation. A total of 3×10^6 ^MSCs in 0.2 ml DMEM were intravenously or intramyocardially administrated 4 weeks after the final subcutaneous ISO-injection. In the case of intramyocardial administration, drug was injected over multiple areas. 

***MSC identification***

Fluorescence-activated cell sorting (FACS) was used to analyze cultured MSCs based on a previous study (30). Trypsinized cells (1×10^6^) were incubated with 5 µg/ml antibodies in PBS at 25 ^°^C in the dark for 15 min. Antibodies used were as follows: CD44-FITC, CD29-PE, CD90–FITC, CD73-PE, CD105-FITC, CD166–PE, CD45-FITC, IgG1-FITC/IgG1-PE, and CD34-PE. Finally, samples were fixed in 1% paraformaldehyde solution and quantitative analyses were carried out using flow cytometer (Partec Pas III, Germany). 

***Assessment of osteogenic*** ***and adipogenic differentiation***

In the case of osteogenic differentiation, MSCs were subjected to the osteogenic induction medium containing DMEM, 10% FBS, 10 mM glycerophosphate disodium, 10^-7^ mM dexamethasone, and 50 mg/ml ascorbic acid for 4 weeks. Von Kossa staining was used to observe calcium deposits. In the case of adipogenic differentiation, cells were incubated with the adipogenic induction medium containing 50 µm indomethacin, 100 µg/ml 3-isobutyl- 1-methylxanthine, 10 µg/ml insulin and 10^-6 ^M dexamethasone for 3 weeks. After washing with PBS, cells were fixed in 10% paraformaldehyde solution at room temperature for 20 min. Oil red O staining was used to evaluate adipogenic differentiation. An invert microscope was used to investigate samples. 

***Assessment of heart function***

*In vivo* two dimensional M-mode echocardiography was carried out using 12 MHz transducer connected to the Vivid 3 Expert ultrasound system 28 days after the final ISO injection and again 28 days after transplantation. Rats were anesthetized with a mixture of ketamine (60 mg/kg) and xylazine (5 mg/kg) to prevent movement and exposed in the supine position. Left ventricular internal dimension at end systole (LVIDs) and left ventricular internal dimension at end diastole (LVIDd) were recorded at the level of the papillary muscle. Echo Pac software (GE Healthcare) was used to analyze data. EF was calculated as follows: EF=(LVIDd^2^ -LVIDs^2^)/LVIDd^2^

***Histological assessment***

Four weeks after transplantation, the animals were sacrificed under deep anesthesia by overdose of ketamine and xylazine; hearts were excised and fixed in 10% phosphate-buffered formalin and embedded in paraffin. The samples were transversely cut into 7 µm thickness sections. 

***Hematoxylin & Eosin (H&E)***

The sections were fixed in 4% paraformaldehyde followed by staining in hematoxylin solution (Boom) and then in eosin solution (BDH Curr Certistain). In the next step, sections were dehydrated and mounted with entellan (Merck).

***Masson’s Trichrome***

The sections were fixed in Bouin’s fixative, and then in 4% paraformaldehyde. In the next step, sections were stained in Weigert’s Hematoxylin, biebrich scarlet acid fuchsin solution, phosphotungstic/phosphomolybdic acid, aniline blue, and subsequently treated with acetic acid before dehydrating and mounting with Entellan (Merck). The percentage of blue staining was calculated using the Image J software (NIH, Bethesda, MD, USA) and defined as fibrotic regions. 

***Statistical analysis***

 All data were expressed as means±SEM. Comparison between three or more groups was performed using one-way ANOVA followed by tukey’s *post hoc* tests. The results were considered statistically significant at *P*<0.05 value. 

## Results

***MSC*** ***characterization***

Four days after initial plating, morphology of cells was similar to fibroblasts. A confluent monolayer of MSCs was formed 16 days after initial plating. 

**Figure 1:**
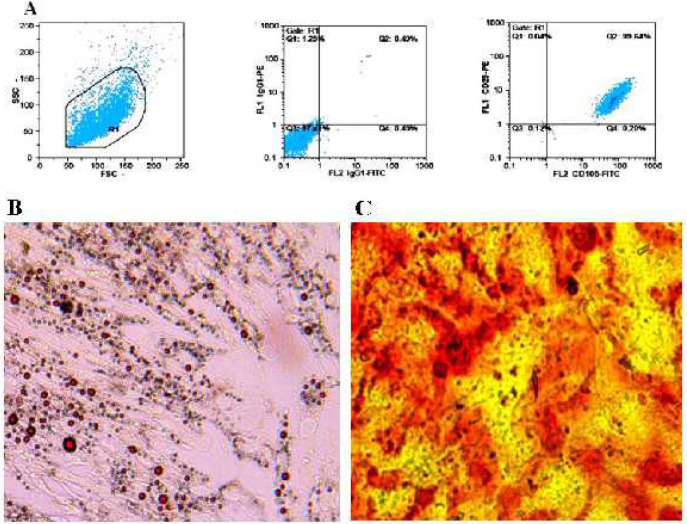
MSC characterization

FACS analysis showed the presence of CD29 in 99.64% of cultured MSCs, whereas CD45 was detected in 1.62% of cells. Likewise, CD34 was found in 3.26% of MSCs, suggesting a highly purified MSC isolation (Figure 1A).

Moreover, oil red O staining and von Kossa staining demonstrated ability of MSCs for differentiation into adipocytes and osteocytes (Figure 1B and 1C).

***Heart function***

**Figure 2:**
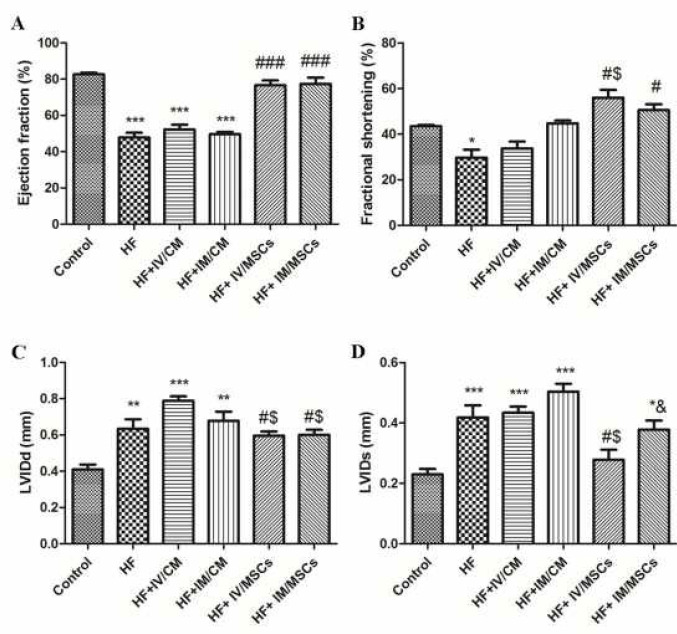
Both intravenous and intramyocardial administration of cultured MSCs significantly improved heart function after HF

As shown in Figure 2A and 2B, EF and fractional shortening (FS) were significantly decreased in HF relative to control. Both intravenous and intramyocardial administrations of cultured MSCs markedly restored EF and FS. There were no significant differences between HF+IM/MSCs and HF+IV/MSCs groups. In addition, a significant increased LVIDd and LVIDs were found in HF compared to control. Both intravenous and intramyocardial administrations of cultured MSCs remarkably blunted increasing of LVIDd, and LVIDs relative to HF, HF+IM/CM, and HF+Iv/CM groups (Figure 2C and 2D). No significant differences were observed among different injection methods. 

***Cardiac fibrosis***

Masson’s trichrome staining was used to evaluate the extent of myocardial fibrosis. Our results showed that ISO-HF resulted in a significant formation of fibrosis. Intravenous or intramyocardial injections of cultured MSCs triggered attenuation of myocardial fibrosis (Figure 3).

***Tissue damage***

Severe tissue damage was found in ISO-HF rats. Intravenous or intramyocardial administrations of cultured MSCs were remarkably decreased tissue damage (Figure 4).

## Discussion

In the present work, a rat model of HF was established to compare cardioprotective effects of intravenous-administered MSCs and intramyocardial-administered MSCs 28 days after final injection of ISO. Some parameters including MSC identification, cardiac function, myocardial fibrosis and ability of MSCs for differentiation into adipocytes and osteocytes were evaluated. Moreover, cardiac morphology or structure was measured in ISO-HF rats treated with intravenous or intramyocardial administered MSCs. Our results showed that both intravenous and intramyocardial administrations of cultured MSCs markedly contributed to the improvement of cardiac function and amelioration of fibrosis. There were no significant differences between the cardioprotective effects of intravenous-administered MSCs and intramyocardial-administered MSCs in ISO-HF rats. ISO-HF is a time-dependent progressive HF with several advantages. These include mimicking of the natural processes of HF and intact coronary circulation, which make it an excellent model to study myocardial fibrosis (31). Researchers have introduced new options for treatment and diagnosis of HF and other degenerative diseases (32-38). Stem cell-based therapies have shown great promise in tissue repair following stroke, HF and ischemia/reperfusion injuries. Many previous studies have shown that intramyocardial injection of MSCs has attracted much attention in recovery of heart function following cardiac injury because cells did not distribute or entrap throughout the body including spleen, liver and lungs (39). Aberrant distribution of MSCs into spleen, liver or lungs results in their differentiation into myofibroblast, fibroblast and alveolar epithelial cells that, in turn, leads to tissue dysfunction (40, 41). The intramyocardial injection of MSCs can improve cardiac function after MI (42). These reports are in agreement with our finding that showed intramyocardial injection of MSCs markedly restored EF, and FS and subsequently blunted the increasing of LVIDd and LVIDs. Likewise, present study demonstrated that the intramyocardial injection of MSCs remarkably prevented cardiac fibrosis. On the other hand, some previous studies have emphasized that intramyocardial injection of MSCs is an invasive and unrepeatable method. These reports have shown that intravenous injections of MSCs have several advantages relative to intramyocardial injection. These include inhibition of prolonged inflammatory process in damaged cardiac tissue, leading cause of adverse cardiac remodeling and ability for repeated injections over time. A recent study indicated that intravenous injection of MSCs not only improves heart function after MI, but also exerts anti-inflammatory effect against excessive inflammatory process in the site of injury (24). Another report indicated that intravenous-administrated MSCs restored heart function via improvement of angiogenesis and myogenesis (43). Moreover, Lim *et al.* reported that intravenous injection of allogeneic umbilical cord-derived multipotent mesenchymal stromal cells led to amelioration of myocardial remodeling in porcine acute MI (44). The authors attributed cardioprotective effects of allogeneic umbilical cord-derived multipotent mesenchymal stromal cells to secretion of paracrine factors in the site of injury (44). In keeping with these findings, our results indicated that intravenous injections of MSCs led to improvement of cardiac function and attenuation of fibrosis. However, both methods markedly contributed to preservation of cardiac structure and morphology. In the present work, we did not observe any significant differences between cardioprotective effects of intravenous and intramyocardial-administrated MSCs after ISO-HF. 

A) Representative FACS analysis, B) oil red O staining, C) von Kossa staining

MSC: Mesenchymal stem cells, FACS: Fluorescence-activated cell sorting

A) EF was significantly reduced in ISO-induced HF rats compared to control (***P<0.001). Intravenous and intramyocardial injection of culture medium did not show a significant change in EF restoration. Both intravenous and intramyocardial injections of cultured MSCs markedly restored EF (###*P<*0.001 compared to HF group, HF+IM/CM and, HF+IV/CM). B) Similar to EF, FS was markedly decreased in HF compared to control (*P<0.05). Intravenous and intramyocardial injection of culture medium did not affect FS restoration. A significant improvement of FS was found in rats treated with intravenous or intramyocardial injections of cultured MSCs (#*P<*0.001 compared to HF group; $*P<*0.05 compared to control). C) LVIDd was remarkably increased in ISO-induced HF rats compared to control (**P<0.01, ***P<0.001). Intravenous and intramyocardial administration of culture medium did not blunt increasing of LVIDd. Intravenous or intramyocardial administration of cultured MSCs significantly reduced LVIDd compared to HF+IV/CM (#*P<*0.05). There was a significant difference in control HF+IM/MSCs and HF+IV/MSCs groups relative to control. D) A significant increased LVIDs was found in ISO-induced HF rats compared to control (***P<0.001). Intravenous and intramyocardial injection of culture medium did not hamper increasing of LVIDs. Intravenous or intramyocardial injections of cultured MSCs significantly blunted increasing of LVIDs (#*P<*0. 01 compared to HF and HF+IV/CM; $*P<*0. 001 compared to HF+ IM/CM; *P<0. 05 compared to HF+IM/CM, & *P<*0. 05 compared to control. There were no statistically significant differences between HF+IM/MSCs and HF+IV/MSCs groups for all parameters

MSC: Mesenchymal stem cells, HF: Heart failure, EF: Ejection fraction, FS: Fractional shortening, ISO: Isoproterenol, IM: Intramyocardial administration, IV: Intravenous administration, CM: Culture medium, LVIDd: Left ventricular internal dimension at end diastole, LVIDs: Left ventricular internal dimension at end systole

**Figure 3:**
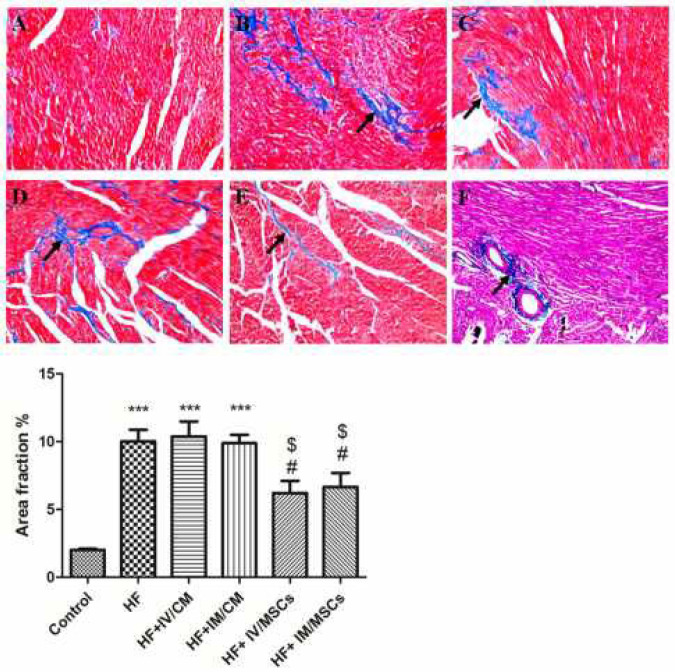
Masson’s trichrome staining showed that both intravenous and intramyocardial injections of cultured MSCs reduced the extent of fibrosis

A) Control. B) HF. C) HF+IV/CM. D) HF+IM/CM. E) HF+IV/MSCs. F) HF+IM/MSCs. A significant formation of fibrosis was observed in HF group relative to control (***P<0.001). Intravenous and intramyocardial injection of culture medium did not show attenuation of fibrosis. Intravenous or intramyocardial administration of cultured MSCs markedly reduced fibrosis (#*P<*0.05 compared to HF, HF+IV/CM, and HF+IM/CM; $ *P<*0.05 compared to control)

MSC: Mesenchymal stem cells, HF: Heart failure, IM: Intramyocardial administration, IV: Intravenous administration, CM: Culture medium

**Figure 3:**
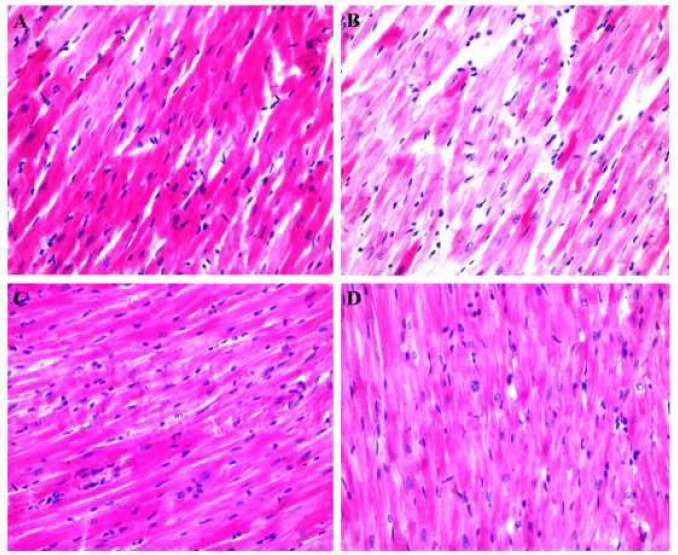
A) Normal cardiac tissue was found in control. B) Severe damage was observed in HF group. Both C) HF+IV/MSCs and D) HF+IM/MSCs remarkably preserved cardiac morphology or structure

MSC: Mesenchymal stem cells, HF: Heart failure, IM: Intramyocardial administration, IV: Intravenous administration, CM: Culture medium

## Conclusion

Our findings demonstrated that both intravenous and intramyocardial-administrated MSCs exert cardioprotective effects against ISO-HF via improvement of heart function and attenuation of fibrosis. Although these results confirm efficiency of both administration methods, this study has many limitations and more future research is needed. Indeed, although stem cell-based therapies have shown safety and efficacy in some clinical and experimental models, their clinical application might be challenged by concerns regarding cell type, cell source, dosage, route of administration, immunogenicity, cell migration from the injection site to other body organs, or their differentiation into undesired cells and deterioration of function of other organs such as lung, liver and spleen. We did not examine possible adverse effects of both methods on other organs such as lung, spleen, and liver.
